# Research on Improving the Partial Discharge Initial Voltage of SiC/EP Composites by Utilizing Filler Surface Modification and Nanointerface Interaction

**DOI:** 10.3390/polym14112297

**Published:** 2022-06-05

**Authors:** Xupeng Song, Wei Yang, Shouchao Huo, Kun Wang, Yuanyuan Wu, Yun Chen, Jian Qiao, Boyang Shen, Xingming Bian

**Affiliations:** 1 State Key Laboratory of Advanced Power Transmission Technology, State Grid Smart Grid Research Institute Co. Ltd., Beijing 102211, China; songxupeng96@163.com (X.S.); 19630100@163.com (W.Y.); wangkun278@163.com (K.W.); yyaeyyae@163.com (Y.C.); buaa_joe@126.com (J.Q.); 2 State Key Laboratory of Alternate Electrical Power System with Renewable Energy Sources, School of Electrical and Electronic Engineering, North China Electric Power University, Beijing 102206, China; huoshouchao@163.com (S.H.); woo15207203196@163.com (Y.W.); 3 Electrical Engineering Division, University of Cambridge, Cambridge CB3 0FA, UK; bs506@cam.ac.uk

**Keywords:** SiC/EP composite materials, partial discharge initial voltage, micro-nano compound, silane coupling agent modification

## Abstract

SiC/EP composites are promising insulating materials due to their high thermal conductivity, stable chemical properties, and nonlinear electrical conductivity. However, the compatibility of micron-sized SiC particles with the organic polymer matrix is poor, and defects such as air gaps may be introduced at the interface, which reduces the partial discharge resistance of the composite materials. In order to improve the partial discharge initial voltage (PDIV) of SiC/EP composites, in this paper, SiC/EP composites with different proportions were prepared by surface modification of filler and compound of micro/nano particles. Firstly, a method of secondary modification of SiC particles was proposed, which was first modified by alkali washing and then silane coupling agent KH560, and the effectiveness of the modification was verified. Therefore, the interface bonding ability between the filler and the matrix was improved, the air gap defects at the interface were reduced, and the PDIV of the composite material was improved. When the filling ratio is 10 wt%, the PDIV was enhanced by 13.75%, and when the filling ratio was further increased, the improvement was reduced. In contrast, the introduction of nanoparticles into the composites can effectively improve the PDIV of composite materials. In this study, nanoparticles were used to form a shell-core structure in epoxy resins to exert their huge specific surface area and active surface properties, thereby changing the overall crosslinking properties of the composites. Through experimental research, the optimal micro-nano particle compounding ratio was explored. Under the optimal mixing ratio, the PDIV of the composite material can be increased by more than 90%.

## 1. Introduction

In high-voltage electrical equipment, such as insulators, cable terminals, transformers, large generators, etc., electric field distortion often occurs in their internal insulation due to their own structural design and operating conditions, resulting in local field strength concentration, which poses a hidden danger to the safe operation of power equipment [1,2,3,4]. Electric field distortion can easily lead to partial discharge in the dielectric. Long-term partial discharge leads to the deterioration and decomposition of the dielectric, and an electrical tree is generated inside the insulating material [5]. As the electrical tree continues to grow, it may cause insulation breakdown or insulation failure, which reduces the service life of equipment, and even cause serious accidents. In order to apply higher voltage levels and improve its partial discharge resistance, it often starts from two aspects: structural design and insulation material modification. In terms of structural design, the installation of shields, pressure equalizing rings and other hardware can make the electric field distribution uniform; the internal electric field distribution can be improved by installing parallel plates inside the insulator, and the local high field strength at the head and end ends can be alleviated. While it is at the expense of increased complexity of fabrication process and increased cost of equipment production; at the same time, it also leads to the introduction of new metal tip burrs or internal air gaps and other insulation defects during the fabrication process. On the other hand, in order to improve the partial discharge resistance of electrical equipment, starting from improving the partial discharge level of the insulating material, by introducing inorganic filler particles, homogenizing the local electric field, and hindering the movement of carriers, the partial discharge resistance of composite materials can be improved. This method can save space for structural design and metal consumables, and at the same time make up for the decrease in insulation level caused by internal bubbles, impurities and other subtle defects in the production process of composite insulating materials [6]. Therefore, it is very necessary to improve the partial discharge resistance in the research on the insulation properties of polymer composites.

Zhe Li, et al. [7,8] used different proportions of micro- and nano-Al_2_O_3_ to fill epoxy resin separately or together, and studied the effects on thermal conductivity, breakdown strength, and resistance to electrical tree growth of composites. The breakdown strength of the composite decreases with the increase of the amount of micro-particles added; the nano-filler can improve the breakdown field strength of the composite under the appropriate filling ratio, and the partial discharge resistance of the nano-composite is stronger than that of the micro-composite. Roy et al. [9] analyzed the difference in the surface state and interfacial bonding ability of micro-scale fillers and nano-scale fillers in polymers. Due to the large curvature of nano-filler particles, the O and H elements on the surface of the nano-filler particles cannot be bonded, but it interacts with the matrix, so its filler/polymer interface bonding effect is stronger. It is believed that the breakdown strength has little to do with the performance of the filler, but mainly depends on the surface properties of the filler and the properties of the interface. The more covalent bonds between the nanoparticles and the matrix, the more conducive to improving the breakdown strength. Fuse N [10] summarized the mechanism of nanoparticle filling to improve the partial discharge performance of composite insulation: the introduction of nanocomposite materials increases the crystallinity; the surface of nanoparticles hinders the movement of gas molecules; the presence of inorganic fillers should not simply considered to be the main mechanism for the resistance improvement, the strong interaction between the resin and nanofillers indicated by the dielectric constant analysis also limits molecular motion. It is believed that the nanoparticles have high surface energy and specific surface area, and can be well combined with the matrix, while the chemical activity of the micro-alumina surface is poor, and a dense structure cannot be formed at the interface.

In summary, the connection between the micron filler particles and the matrix is poor, and a large number of defects are generated at the interface, which is easy to form a channel for the development of partial discharge, resulting in the deterioration of the partial discharge resistance and breakdown characteristics of the composite material. However, due to the small radius of nano-filler particles, the scale effect is produced, and its impact mechanism on composites is very different from that of micro-composites [5,7,11]. Nanoparticles with large specific surface area and many surface-active groups can form bonds with epoxy resin molecules, making the interface tightly bound, introducing trap density and reducing carrier mobility. As a result, the partial discharge resistance and electrical strength of the composites were significantly improved [8,12]. Therefore, in terms of improving the PDIV of composite insulating materials, feasible research methods can include the following two points: using the interface effect of nanoparticles to build a shell-core structure, so that the movement of carriers inside the composite material could be hindered; modifying the surface of micro-particles to improve the adhesion of particles to the matrix.

Silicon carbide (SiC) inorganic fillers have excellent properties such as stable chemical properties, high thermal conductivity, compact structure, high hardness, and wear resistance. Filling SiC can significantly improve the thermal conductivity of composite materials. At the same time, the composite dielectric filled with SiC has nonlinear conductivity characteristics [13,14]. When the local electric field in the insulating medium is distorted, with excessively high field strength introduced, the conductivity of the dielectric in the corresponding region increases, and the space charge is diffused, which can play the role of homogenizing the electric field [15,16]. The low cost of SiC is conducive to industrial application and promotion. Therefore, SiC/EP has broad application prospects in the packaging of large-capacity power devices and the insulation of high-voltage electrical equipment [17]. When a small amount of nano-SiC particles are introduced into the micro-SiC/EP composite, it will have a significant impact on the degree of cross-linking of the composite matrix and the internal air gap defects [18]. The results show that nanoparticles can improve the thermal stability of the system by enhancing the crosslinking in the polymer. At the same time, it tends to act as a filler for voids, reducing the void content of the composite material, which translates into enhanced mechanical properties and improves the overall mechanical properties. Chi et al. [19] prepared SiC/EP composites, which exhibited nonlinear conductance properties under the action of an electric field. When the filling ratio of SiC is too large, the breakdown field strength of the composite material decreases seriously, and the method of compounding other reinforcing fillers can improve it. The inorganic microparticle fillers and the polymer matrix are incompatible with each other, and there is almost no chemical bond. Through the method of organic modification on the surface of the filler, a layer of organic molecules is attached to the surface of the inorganic filler, and then reacts with the matrix polymer during the cross-linking and curing process to improve the compatibility between the filler and the matrix and reduce the air gap or other defects at the interface. Tanaka et al. [20] prepared SiC/EP composites by using SiC particles with a diameter of 50 nm and investigated the effect of PD erosion. The study found that filling nano-SiC particles can effectively improve the ability of the composite to resist partial discharge erosion, and there is an optimal proportion of the filling amount. The filling of nano-silicon carbide also has a positive effect on improving the creeping partial discharge initial voltage of epoxy resin matrix composites [21]. Kim et al. [22] made SiC magnetically responsive by introducing strong paramagnetic iron oxide (Fe_3_O_4_) nanospheres, and fabricated vertically aligned silicon carbide (SiC)/epoxy composites using a magnetic field along the heat transfer direction. In order to better generate iron oxide on the surface of SiC, hydrofluoric acid (HF) was used to remove SiO_2_ adsorbed on the surface of SiC particles, and hydrogen peroxide solution was used to heat to introduce oxygen-containing functional groups. Gu [23] et al. used titanate coupling agent NDZ-105 to modify the surface of nano-β-SiC whiskers, and prepared β-SiC/nitrocellulose plastic composites by powder blending method. The results show that: NDZ-105 forms chemical bonds with β-SiC whiskers and forms an organic monolayer, so the tensile, bending and impact properties of the composites are significantly improved. Gu [24] has also used KH560 silane coupling agent to modify the surfaces of β-SiC partlcles with a diameter of 1–2 μm, and analyzed the effectiveness of the modification by the tests of infrared spectroscopy (FTIR) and thermal weight loss (TGA). The mechanism of the modification is analyzed, but the research mainly focuses on the change of the mechanical properties of the material. The ultimate goal of modifying the surface of SiC particles by inorganic [25] or organic [26] is to improve the degree of bonding between SiC particles and the matrix, so as to improve the dispersion of particles in the matrix and reduce interface defects, thereby improving the electrical properties of the composite, thermal and mechanical properties.

In the research of epoxy resin-based filled composite insulating materials, in order to improve the electrical and thermal properties of the composite materials, SiC/EP is a good choice. However, although the direct filling of SiC micro-particles can greatly improve the thermal properties of the composite, the surface of the micro-SiC particle is smooth and lacks groups that could bond with EP, which will lead to air gap defects at the interface between the particles and the matrix. In order to solve the problems such as the decrease of electrical strength and the decrease of the initial voltage of partial discharge caused by the filling of micron SiC particles, two optimization methods were adopted in this study. On the one hand, doping nano-particles in composites partially replaces micro-particles to exert its interface scale effect, forming nano-interfaces in the composites, reducing the carrier mobility and improving the electrical strength. The key is to obtain the best compounding ratio through experiments, and to obtain the general trend of change. On the other hand, the method of surface modification of SiC particles is studied in this paper to solve the problem that the surface of SiC particles is difficult to combine with the matrix. The method of alkaline washing and secondary modification of coupling agent is proposed, and the effective effect of modification is verified by macroscopic and microscopic tests, which provides an effective method for surface modification of SiC microparticles.

## 2. Materials and Methods

### 2.1. Experimental Materials

Epoxy resin (EP) has excellent bonding, anti-corrosion, molding and thermal stability properties, and is superior in terms of mechanics, thermal stability, insulation ability and chemical resistance. They can therefore be used as coatings, adhesives and molding materials, and are widely used in electrical and electronic applications. Epoxy resins are also liquid before curing and have good flowability, allowing for easy preparation of filled composites and their modification. There are many types of epoxy resins. Bisphenol-A epoxy resins have high electrical strength and superior insulation properties and are widely used in electrical engineering. Liquid bisphenol A epoxy resins are often used for casting and impregnation in the field of electrical insulation, while solid resins are mainly used for insulation encapsulation, equipment insulation, etc. In this paper, Bisphenol A type epoxy resin is chosen as the base material.

Epoxy resin in the curing process, its cross-linked network of molecular weight, morphology and cross-link density are different due to the curing agent and significant changes, and thus the cured products of electrical, chemical and mechanical properties are also very different. Acid anhydride curing agent is characterized by low skin irritation, long service life, its curing reaction with epoxy resin and the resulting composite material has excellent performance, especially the excellent dielectric properties, so mainly used in the field of electrical insulation. Therefore, in the choice of epoxy resin curing agent, acid anhydride curing agent was selected. Silicon carbide (SiC) inorganic filler has significant advantages such as chemical stability, high thermal conductivity, high hardness, low cost, etc. It is also widely used in the packaging and insulation of high voltage devices due to its non-linear electrical conductivity characteristics. In this paper, SiC is selected as a filler and the role of micron and nanoparticle size SiC particles in the composite is also investigated to improve the partial discharge resistance of composites. The experimental materials used in the experiments are listed in Table 1.

### 2.2. Experimental Equipment

The experimental preparation of filled composites is carried out using high-speed stirring paddles, constant temperature oil baths, electronic balances, magnetic stirrers, vacuum ovens, centrifuges and other equipment. The performance characterization mainly uses a dielectric spectrometer, breakdown voltage tester, infrared spectrometer, density meter, etc. The experimental test equipment required for this experiment is listed in Table 2.

### 2.3. Experimental Sample Sample Preparation

#### 2.3.1. Preparation of SiC/EP Composites

The particle size of silicon carbide particles is small, the chemical state is stable, and the surface of the particles is not easy to react with polymer composite materials, so the problem of particle agglomeration is prone to occur in the process of SiC/EP prepareation. In the preparation, the filler particles should be uniformly dispersed in the matrix. The preparation process is as follows:

(1) According to the calculated proportion, weigh the liquid epoxy resin in a beaker, heat it by the oven to 60 °C to improve its fluidity, and then pour it into a three-necked flask and heat it in an oil bath at 60 °C.
(2) Weigh the curing agent with a mass ratio of liquid epoxy resin and curing agent of 100:85, pour it into the flask, and mix it with the preheated epoxy resin.
(3) Weigh the SiC filler powder according to the experimental calculation ratio, add it into the mixed solution in the three-necked flask, stir at a high speed in an oil bath at 60 °C to disperse the filler evenly, set the rotation speed to 360 r/min and continue 60 min;
(4) After the epoxy resin, curing agent and filler are fully mixed, add the accelerator dropwise at a mass ratio of epoxy resin and accelerator of 100:1, and continue to stir at a constant speed in an oil bath at 60 °C, with a rotation speed of 260 r/min, time of 10 min;
(5) Pour the mixed liquid into a beaker, put it into a vacuum oven preheated to 60 °C, and perform a vacuuming operation. Maintain the temperature and repeat the extraction several times until the mixed liquid in the beaker has no obvious bubbles overflowing;
(6) Spray the mold release agent on the inner surface of the mold and put it into a blast oven for preheating and drying treatment. The preheating temperature is 100 °C until the inner surface is dry. The mixed liquid was taken out of the vacuum oven and poured into the mold, and then put into a blast oven for curing and cross-linking reaction. The heating curve was 100 °C for 4 h, and then turned to 150 °C for 10 h. After the solidification is completed, it is naturally cooled to room temperature and demolded to obtain the sample to be tested.

In order to study the influence of micro-nano SiC particles on the electrical properties of composites under different filling ratios, the total filling amount of SiC was kept unchanged, and nanoparticles were introduced to replace part of the micro-particles, thereby changing the ratio of the two. The ratio of micro-nano particles of each prepared sample is shown in Table 3, in the following discussion in this article, the composite materials were indicated by the serial numbers. For example: 19M/1N/EP represents the epoxy composites filled with 19 wt% microparticles and 1 wt% nanoparticles.

#### 2.3.2. SiC Particle Surface Coupling Agent Modification

When directly modifying SiC with a surface coupling agent, it is difficult to react due to the stable chemical properties of SiC. It is found that the number of coupling agent groups on the surface of SiC powders after direct modification is rare. In order to increase the number of surface groups, it is first necessary to increase the number of hydroxyl groups on the surface of SiC. There are impurities such as Ca, Fe, and Mn on the surface of SiC during the production process. The combination of these high-valent metal cations with silanol groups [Si-OH] will reduce the number of active hydroxyl groups on the surface of SiC particles, which can be removed by alkali washing or acid washing. In the research process, acid washing and alkali washing were tried respectively. It was found by infrared spectrum that the modification effect was better when alkali washing method was adopted and coupling agent was used to modify. Therefore, the secondary modification method of coupling agent modification after alkali washing to remove impurities was selected in this paper. In addition, there will be Si-O-Si bonds and Si-O-C bonds on the surface of silicon carbide due to oxidation, which can be hydrolyzed to generate silanols under strong alkaline conditions by alkaline solution treatment. Silanol is a highly active group, which has a great effect on the next surface modification. The steps of alkaline washing modification are relatively simple:

(1) Prepare 2 mol/L NaOH solution;
(2) Mix 50 g of SiC particles with a particle size of 1.5 μm with 200 mL of NaOH solution, and stir at 40 °C for 2 h;
(3) Centrifuge the solution and wash it with deionized water several times until the pH value of the solution is 7, and dry after the last centrifugation.

The dry and ground SiC powder after alkaline washing modification is used for secondary modification with silane coupling agent KH-560. The modification steps of the silane coupling agent are as follows:

(1) Prepare 400 mL of solution according to the volume ratio of ethanol to ultrapure water of 19:1, pour it into a flask, weigh 50 g of SiC particles dried at 80 °C for 6 h, add them to the flask, and stir at a constant temperature of 60 °C for 10 min to make the SiC particles fully dispersed in solvent;
(2) Drop 2.5 g of silane coupling agent KH-560 into the SiC/ethanol/water solution, and stir at a constant temperature of 60 °C for 6 h to fully react;
(3) Pour the solution into a plastic reagent bottle, use a centrifuge to separate the SiC particles from the solvent, take it out, dry it at 50 °C for 8 h, and grind it with an agate mortar for use.

**Figure 1:**
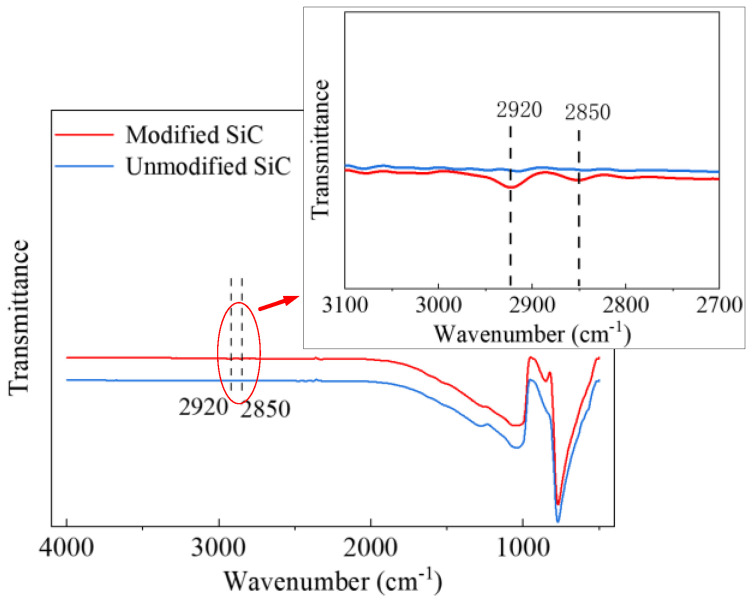
Fourier infrared spectra of unmodified and secondary modified SiC powder.

The infrared spectrum of the SiC powder materials before and after modification was measured as shown in Figure 1. The wavenumbers of 2920 cm^−1^ and 2850 cm^−1^ correspond to methylene groups were discovered, which represent the introduction of silane coupling agent. Compared with the absorption peak of SiC powder itself, the absorption peak of surface functional groups has a smaller amplitude, so it is necessary to enlarge the infrared spectrum. The coupling agent molecules react with the hydroxyl groups on the SiC surface. After centrifugation, washing and drying, the coupling agent molecules can still be attached to the surface of SiC powder particles.

### 2.4. Preparation of Samples for Partial Discharge Testing

#### 2.4.1. Equipment for Partial Discharge Testing

The equipments of partial discharge tests are listed in Table 4.

#### 2.4.2. Test Circuit and Experiment Platform

**Figure 2:**
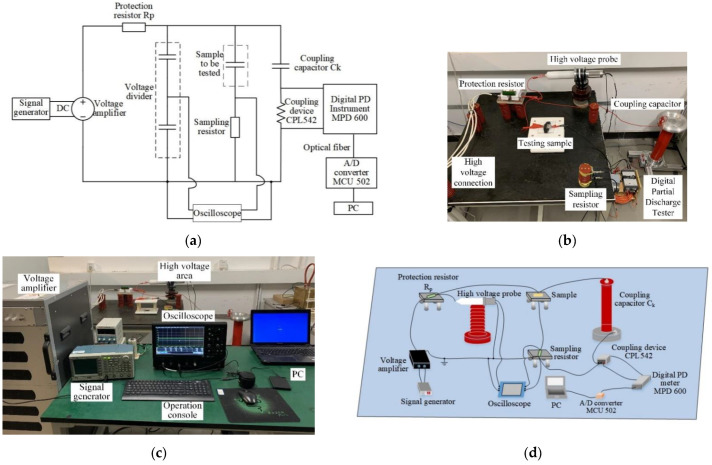
(**a**) Partial discharge test measuring circuit; (**b**) Schematic diagram of the experimental platform; (**c**) Schematic diagram of the construction of experimental circuit; (**d**) Schematic diagram of the experimental platform.

According to IEC 60,270 standard, the test circuit and experiment platform were built. The test circuit is shown in the Figure 2a.

In this paper, the method of pulse current was used to measure partial discharge. Figure 2c shows the circuit topology used for partial discharge measurement. The equipment used in the tested loop includes a signal generator, a voltage amplifier, a high voltage protection resistor, a voltage divider, a coupling capacitor, a sampling resistor, a detection impedance, a digital PD meter, an analog-to-digital converter, and a PC.

The signal source is sent out by the signal generator, and the voltage amplifier amplifies the signal source according to the ratio of 4000:1 to obtain the DC high voltage. By changing the size of the signal source, the DC voltage can be proportionally expanded. Compared with the DC high-voltage source adjusted by the knob, the combination of the signal source and the voltage amplifier makes the voltage size adjustment more accurate and flexible. When a partial discharge occurs inside the solid insulating material, a high-frequency pulse current is generated. The discharge pulse signal can be obtained by measuring the voltage fluctuation of the high voltage end of coupling capacitor with a partial discharge meter.

The value of the protection resistor is selected as 3 MΩ to prevent the high current which caused by the breakdown of sample or line insulation from damaging the power supply or measuring equipment. At the same time, it can play the role of DC filtering under normal operation. The value of the coupling capacitor is 800 pF, and the rated voltage is 50 kV. The value of the coupling capacitor should be selected to be large enough to obtain high-frequency partial discharge pulses. On the low-voltage outlet side of the capacitor, the impedance measurement device CPL 542 is connected in series, and its interior is composed of RLC circuit. The frequency range of partial discharge signal that can be detected is 20 kHz–6 MHz. Connecting the digital partial discharge instrument in parallel to both ends of the detection impedance can prevent the partial discharge instrument from being damaged when the current in the loop is too large. MPD 600 partial discharge instrument is manufactured by Austria OMICRON company, suitable for laboratory measurement of partial discharge signal of high-voltage electrical equipment, the center frequency is 32 MHz, the input partial discharge pulse frequency range is 0–20 MHz, PD event time resolution is less than 2 ns, the noise of system is less than 0.015 pC, and the measurement accuracy of the partial discharge signal is ±2% of the standard PD value. The output end of MPD 600 partial discharge instrument sends measured partial discharge signal to the MCU 502 optical fiber controller through the optical fiber. The optical signal is converted to a digital signal after an analog-to-digital converter, and then transferred to a personal computer using a USB interface. The adoption of optical fiber signal transmission can reduce the interference of environmental electromagnetic field on the transmission line in the transmission process, further restrain background noise, and also realize electrical isolation of test loop and PC end.

The signal generator (Tektronix AFG3022C) can output square wave, sine wave, pulse, DC and other signals. This paper mainly used DC signal, and its output voltage peak range is 10 mV~20 V. By adjusting the knob, each turn can change the increase or decrease of 10 mV.

The voltage amplifier (Matsusada AMPS) adopts the amplification gain of 4000:1. This paper adopts DC signal input and DC voltage output.

In order to ensure the accuracy of the voltage applied to high-voltage terminal, a high-voltage probe and an oscilloscope are used to measure the high-voltage DC. The high voltage probe (North star PVM-4) uses a 1000:1 ratio to downscale the signal and input it to the oscilloscope for display. The maximum DC voltage that the PVM-4 can measure is 40 kV, the fastest rising edge time is 2.5 ns, and the error level can be maintained within 5% at frequencies above 5 MHz.

A 300 Ω sampling resistor is connected in series with the ground of sample to connect the measurement signal to oscilloscope. Taking this as an auxiliary measurement method of partial discharge pulse signal, its measurement accuracy and sensitivity are not as good as that of digital partial discharge instrument, but it can play the role of auxiliary judgment.

### 2.5. Test Method of Partial Discharge Initial Voltage

#### 2.5.1. Preparation of Samples

In order to simulate the severe electric field distortion that polymer insulating materials need to face under actual working conditions, a needle-plate electrode is used to construct an extremely non-uniform electric field. Epoxy resin is generally used as an insulating encapsulation material to isolate high-voltage electrodes or electrical appliances from the external environment. Therefore, in this paper, the needle electrode is poured inside the epoxy resin composite material to simulate the partial discharge characteristics under encapsulation conditions.

The relative position of high-voltage terminal needle electrode and ground terminal plate electrode will significantly affect the electric field distribution in the space. In order to ensure the comparability of measurement results, it is necessary to ensure that the electric field constructed in each test sample is consistent, which includes the relative perpendicular of needle electrode and plate electrode, and the consistent distance between the needle electrode and plate electrode.

**Figure 3:**
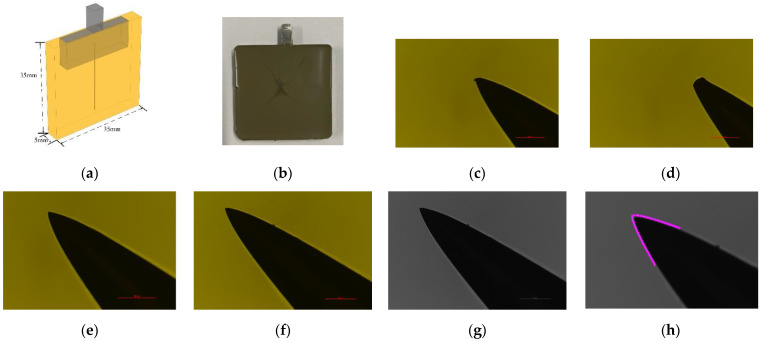
(**a**) Schematic diagram of sample size and electrod arrangement for PD testing; (**b**) Schematic diagram of PD sample; (**c**) Microscope picture of distorted needle tip; (**d**) Microscope picture of blunt needle tip; (**e**) Needle electrode with symmetrical shape and rounded tip; (**f**) Original image of needle tip; (**g**) Grayscale image in 8-bit of needle tip; (**h**) The contour of needle tip.

In this paper, a metal tool is placed at the high voltage end and the needle electrode is inserted into a pre-drilled hole to ensure that the direction of needle electrode is vertical. The needle electrode is cut from the tail to change the length, so that the length of the needle electrode after it is inserted into the hardware remains the same, and the distance from the end of the needle to the bottom of the mold is 3 mm. The schematic diagram of partial discharge sample and embedded needle electrode is shown in Figure 3a,b.

Pre-embedded needle electrodes are stainless steel acupuncture needles, which are made by cutting to length, and their toughness, hardness, and uniformity of processing technology can meet the needs of experiments. Acupuncture needles have been used in many studies.

The radius of curvature and shape of needle electrode affect the initial discharge voltage and development process of partial discharge within the composite material. In order to ensure the comparability and scientificity of measurement result, it is necessary to screen the needle electrode and measure the radius of curvature of needle electrode to characterize the radius of curvature distribution of the needle electrode.

The tip curvature radius of needle electrode is in the order of micrometers. At this order of magnitude, the shape of needle tip is easily damaged during processing and transportation, resulting in processing errors and impacts. Therefore, the needle electrode should be initially screened first. The needle electrode is placed under the objective lens, and the magnification of eyepiece is 50*10 times. By adjusting upper and lower positions of the stage, a clear focus is achieved, and then the backlight brightness is adjusted until a clear and bright image of the needle tip is observed. By using the scale bar matched with microscope equipment, the magnification in image is calibrated, so that the obtained image size data is accurate. By using microscopic image processing software “NIS-Elements” to take pictures, save them, and mark the pictures with the corresponding needle electrodes for subsequent screening and use.

By observing the microscope pictures, the needle tip electrodes with obvious defects were screened out, as shown in Figure 3c,d:

The tip curvature radius of the symmetrically shaped, rounded tip needle electrode was measured and characterized.

The commonly used measurement method is simple but low in accuracy: drawing a coincident circle at the position of the needle tip, and the radius of the circle is the radius of curvature of the needle tip. Although this method is intuitive and fast, it is affected by the judgment error of human eyes, and in some cases, it cannot make the ball closely fit the needle tip. The method used in this paper is: use the “Kappa” plug-in of “ImageJ” software to process the captured pictures, and automatically extract the curvature value of the contour line of the needle tip.

ImageJ is an open source image processing software developed by USA National Institutes of Health (NIH) based on java, which supports image processing plug-ins with different functions proposed by other research institutions. Users can choose the corresponding function plug-ins to use according to their needs. This article uses the “Kappa” plug-in made by Hadrien Marya and Gary J. Brouhard of McGill University in Canada, which can implement functions such as curve fitting and curvature measurement.

The measurement process of the tip curvature radius is as follows:

Firstly, the captured needle tip image is imported into ImageJ, and the image is taken as a grayscale image, so that the software can automatically identify the needle tip outline according to the color difference, as shown in Figure 3e–g.

The length scale has been marked in the captured picture, and the unit is micrometer. In ImageJ, the image is in pixels, so it is necessary to use the set scale function of ImageJ to convert the scale length in the picture to the number of pixels.

Subsequently, the grayscale image of the needle tip image was imported into the Kappa plugin, and points were manually taken along the contour to determine the solution area for curve fitting. After the error limit is determined, the software will automatically select points to fit according to the color difference in the solution area, and obtain the fitting curve along the needle tip profile, as shown in Figure 3h. The fitting curve adopts the cubic spline interpolation method, and the fitting curve can fit the contour of the original image well, which is more accurate than the “equivalent small circle method”. The curvature at each point of the fitted curve is derived, and the inverse of the curvature is calculated to obtain the radius of curvature. The minimum value of the radius of curvature corresponds to the radius of curvature of needle tip.

**Figure 4:**
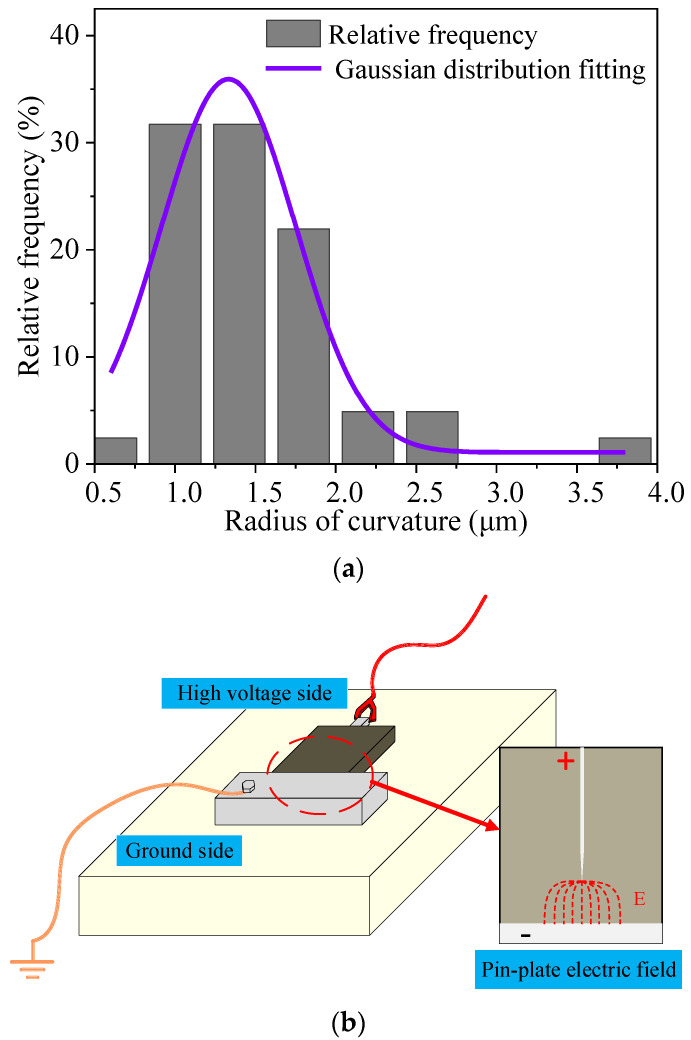
(**a**) Radius distribution of needle tip curvature; (**b**) Pressurization of PD samples.

The distribution of the radius of curvature values for the needle electrodes is shown in Figure 4a.

Most of the needle tip curvature radius is concentrated between 1–1.5 μm, and the needle electrode with a curvature radius of 1.3 ± 0.1 μm is selected as the experimental electrode. Repeated experiments were carried out in order to reduce the effect of the difference in the radius of curvature on the experimental results of the PDIV.

A metal plate electrode for grounding was fixed under the prepared pre-embedded needle electrode PD sample, and a needle-plate electric field was constructed inside the composite material. The compression method of the composite material sample is shown in Figure 4b.

#### 2.5.2. Test Method for Partial Discharge

First, the partial discharge test circuit was verified. According to the preliminary measurement results, the partial discharge initial voltage of the samples studied in this paper is generally not greater than 16 kV. Therefore, under no-load conditions, a DC voltage is applied to the loop until 20 kV to determine whether there is a pulse interference signal and determine the background noise level under no-load conditions. When local defects are generated in the equipment such as coupling capacitors and protection resistors, partial discharge pulse signals will also be generated under high voltage, so these interferences need to be eliminated first. After measurement, the background noise is not more than 2 pC, in line with the IEC 60270 measurement standard.

Then, the discharge capacity of the partial discharge meter was calibrated, and the CAL 542 charge calibrator was connected to the test circuit shown in Figure 2, Figure 3 and Figure 4 to replace the sample to be tested. It generates charges of 50 pC, 100 pC, and 200 pC respectively, calibrates the reading of the partial discharge meter, and judges the accuracy and sensitivity of the test loop.

Finally, the PDIV was measured. According to the definition of GB/T7354 and IEC 60270, a voltage significantly lower than the expected value of the initial voltage is applied to the test sample, and the voltage is gradually increased until the discharge occurs. The test voltage at this time is the partial discharge initial voltage. In IEC 60270, there is no clear regulation on the initial partial discharge initial voltage of DC partial discharge. In the current research, the voltage when the discharge pulse can be continuously generated after the first partial discharge pulse is generated is generally used as the DC partial discharge initial voltage. In this paper, the step-up boosting method is adopted, and the duration of each voltage segment is maintained for 5 min to fully judge whether partial discharge occurs. The boosting rate between each voltage segment is 0.04 kV/s, which corresponds to one grid of the signal generator. This facilitates accurate control, and the boost can be stopped in time after the PD pulse occurs. Each group of samples was measured 5 times, and the average value was taken as the partial discharge initial voltage.

## 3. Experimental Results and Discussion

### 3.1. Partial Discharge Initial Voltage of SiC/EP Composites

**Figure 5:**
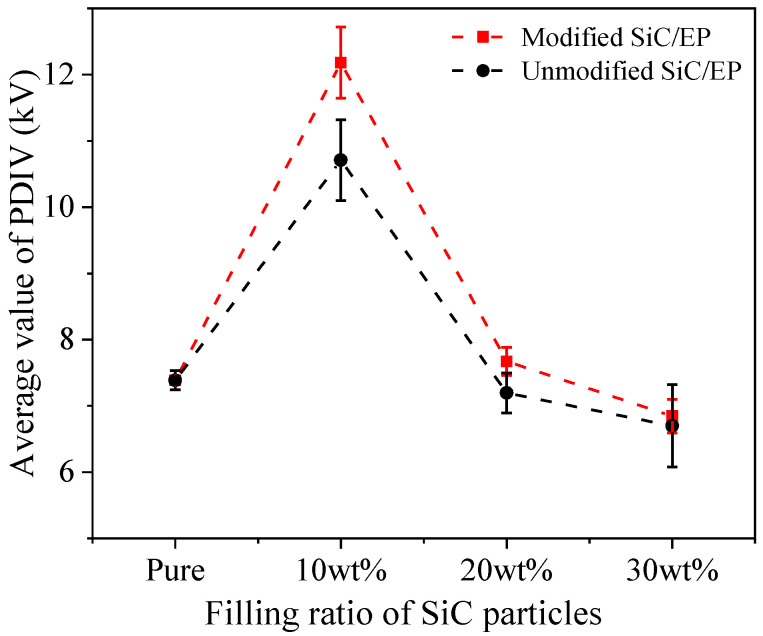
Partial discharge initial voltage of SiC/EP composites.

By the test method described above, the PDIV of the secondary modified SiC/EP composite is shown in Figure 5.

It is showed that with the increase of SiC filling ratio, the partial discharge initial voltage (PDIV) of composite materials increased first and then decreased. When the total filling amount is 10 wt%, the PDIV increased by 44.94%. With further increase in filling ratio, PDIV decreased rapidly. When the filling amount was not large, the filler was isolated and dispersed inside the matrix, the filler had little distortion to the electric field, and would not cause agglomeration to introduce large void defects. The carriers moved directionally under the action of the electric field, passing through the epoxy, two-phase interface, and SiC particles in turn. In the process of motion, the trap barrier generated by the interface needed to be crossed, the average energy was decreased, and a higher electric field was required to stimulate impact ionization. When the filling ratio increased, the distortion effect of the fillers on the electric field was enhanced, and the fillers overlapped each other to form a coherent air gap passage. The ionization occurred first at the particle interface, and further developed along the coherent air gap defect and the PDIV of the composite decreased. After the surface modification of SiC filler, the PDIV of composite materials were improved. When the filling ratio was 10 wt%, the partial discharge initial voltage of the surface-modified SiC/EP is increased by 13.75%. It can be seen from the variation law of the measured values that when the filling ratio of the filler increases, the effect of increasing the partial discharge initial voltage of composite materials becomes weaker.

**Figure 6:**
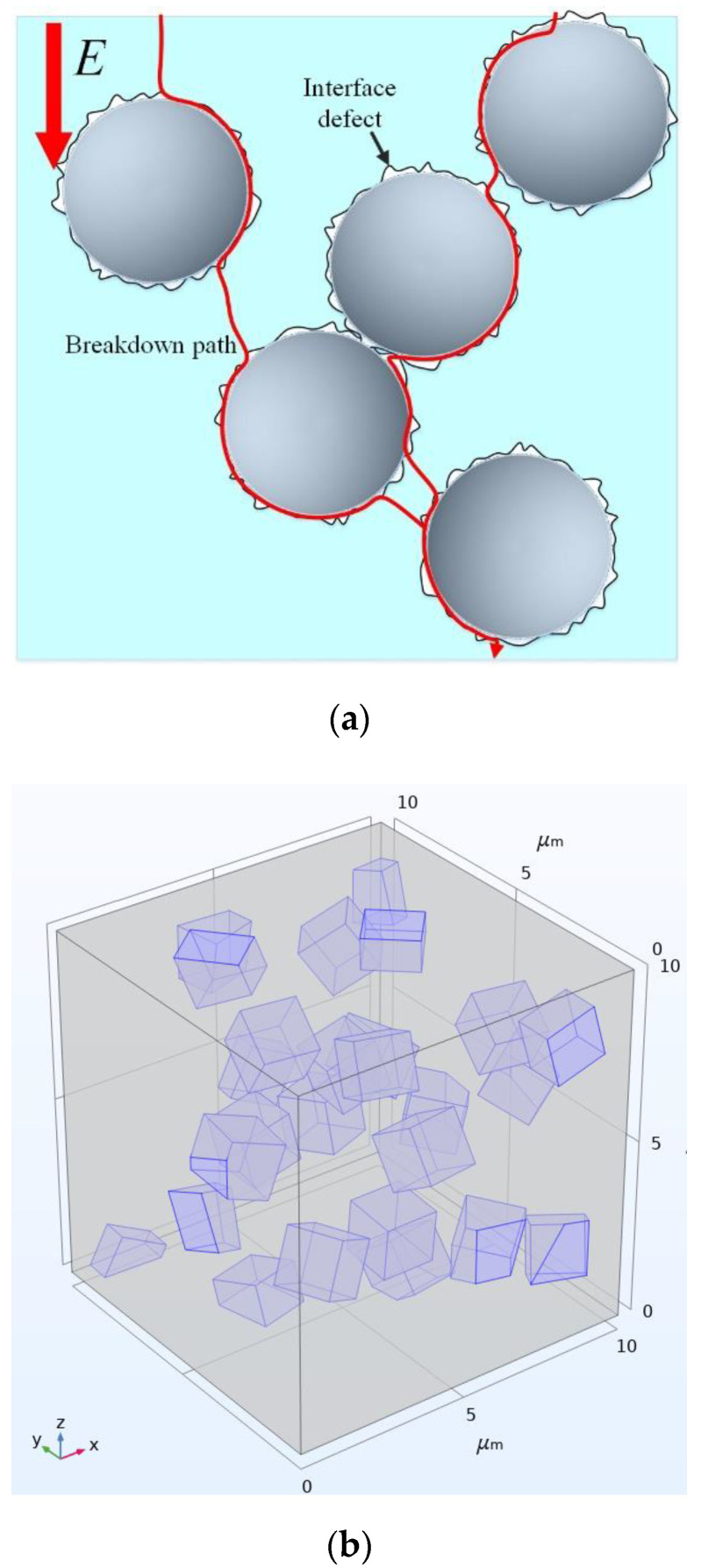

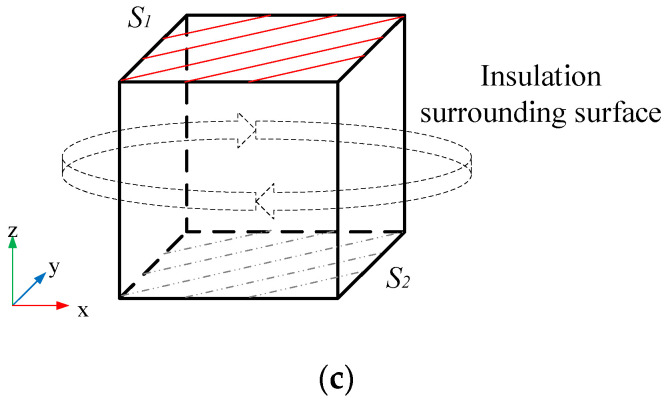
(**a**) Schematic diagram of micro-filler/matrix interface defects; (**b**) RVE model of composite material; (**c**) Schematic diagram of boundary condition setting.

As shown in Figure 6a, after the micro-filler was introduced into the composite materials, the electric strength of the air gap at the interface of the micro-filler was low and the electric field strength was high. On the other hand, since the electrical conductivity and relative permittivity of micro-SiC were much larger than those of the epoxy matrix, the filler would cause electric field distortion inside the composite materials, which would make the epoxy interlayer between the filler particles subject more electric field strength. Therefore, the problems of electrical breakdown and partial discharge were more likely to occur in this situation.

The random adsorption sequence method was used to establish a representative volume element (RVE) model of the random distribution of filler particles, and the influence of the internal electric field distribution of composites after filling with SiC particles was simulated and analyzed.

It can be seen from the SEM pictures of the composites that the SiC particles used in this paper are angular, mostly cubic and irregular in shape.

In order to simplify the calculation without losing generality, the filler particles in the simulation calculation are cubic, and the side length is set to 1.5 μm; the representative volume element (RVE) is set to a cubic area with a side length of 10 μm to contain enough microscopic information. The random adsorption sequence algorithm was used to determine the position and angle of the filler particles, and randomly distributed filler particles were generated inside the RVE. As shown in Figure 6b, according to the density of SiC (3.18 g/cm^3^) and the measured density of pure epoxy resin after curing (1.2 g/cm^3^), a representative volume element (RVE) model of the composite material under 10 wt%, 20 wt%, and 30 wt% filling was established, respectively.

The conductivity of epoxy resin tends to show nonlinear characteristics under strong electric field, and the conductivity of silicon carbide also shows nonlinear characteristics related to the electric field. The measured conductivity data were fitted nonlinearly to obtain conductivity parameters. The relative dielectric constant of epoxy resin was set to 2.3 and the relative dielectric constant of SiC was set to 10 at a frequency of 50 Hz. The parameters are shown in Table 5.

In the experimental test of breakdown voltage, according to the IEC test standard, the electric field of the composite material increases from 0 kV/mm until it reaches the breakdown, and the breakdown field strength of the SiC/EP composite materials is generally greater than 10 kV/mm, so the composite material is assumed to withstand an electric field of 5 kV/mm in the simulation. In the RVE model with a side length of 10 μm, the voltage difference between the upper and lower surfaces is 50 V. The boundary conditions are set as shown in Figure 6c.

The boundary conditions are set as Formula (1)

(1) $$\left\{\begin{array}{c} {\left.\frac{\partial V}{\partial n}\right|}_{\Gamma }=0\\ {\left.V\right|}_{S_{1}}=V(t)\\ {\left.V\right|}_{S2}=0 \end{array}\right.$$

In the formula, Γ represents the side surface around the RVE; *S*_1_ represents the upper surface; *S*_2_ refers to the upper surface; *V(t)* represents the AC voltage (V) applied to the upper surface, *V(t)* = 50sin(100πt).

In order to solve the internal electric field intensity of the composite material, under the action of the AC electric field, the governing equations should include the Poisson equation, the current continuity equation, the current density equation and the constitutive relationship equation. The governing equation is as in Formula (2)

(2) $$\left\{\begin{array}{c} E=-\nabla \varphi \\ \nabla \cdot J=0\\ J=\sigma E+\frac{\partial D}{\partial t}\\ J_{c}=\sigma E\\ J_{d}=\varepsilon_{0}\varepsilon_{r}\frac{\partial E}{\partial t} \end{array}\right.$$

where *E* represents electric field strength (V/m); *φ* represents electric potential (V); represents conductivity (S/m); *J_c_* represents conduction current density (A/m^2^); *J_d_* represents displacement current density (A/m^2^); *D* represents the electric displacement vector (C/m^2^).

**Figure 7:**
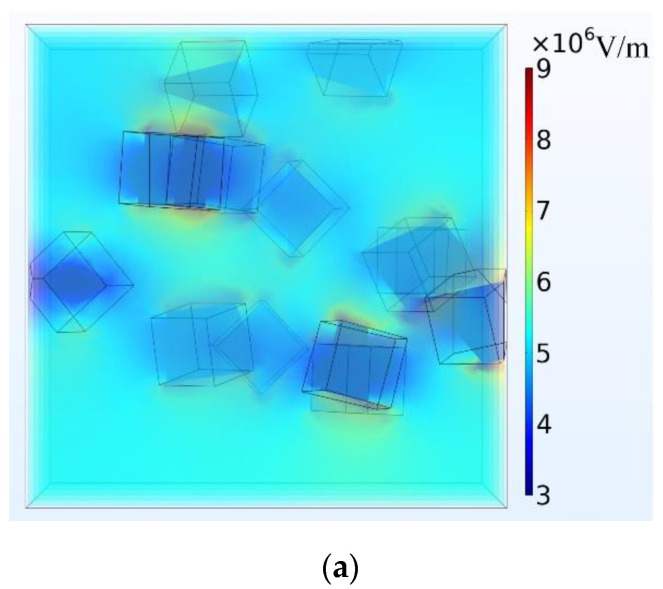

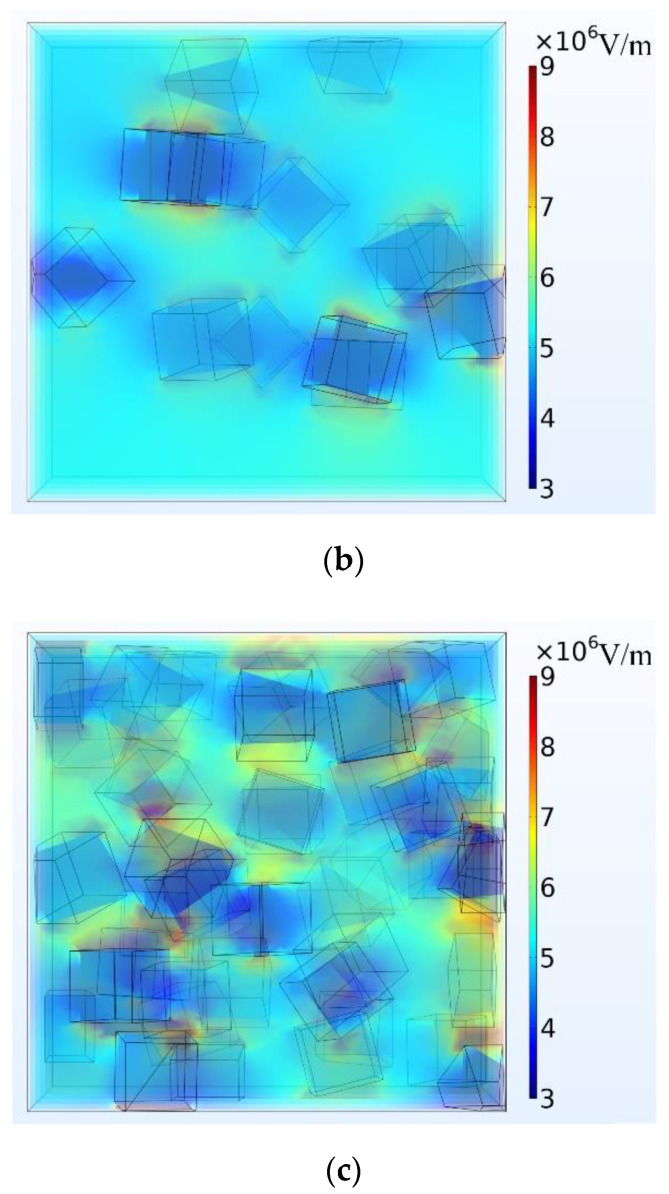
Cloud diagram of electric field distribution inside the composite material; (**a**) 10 wt% composite model; (**b**) 20 wt% composite model; (**c**) 30 wt% composite model.

The cloud diagram of the electric field distribution inside the composite material under different filling ratios is shown in Figure 7.

Since SiC particles have higher electrical conductivity and dielectric constant than those of the matrix, the external electric field will affect the distribution of the electric field inside the material. As can be seen from Figure 7, the electric field is seriously distorted at the location where two particles are close, resulting in a local high field strength of the epoxy resin matrix in this part [27]. With the increase of the filling ratio of SiC particles, the average geometric distance between SiC particles decreases, the degree of electric field distortion is more serious, there are more places with local high field strength, and the field strength value of part of the epoxy matrix is also higher. At the same time, in the actual experiment, there are often air gap defects between the micro-particle filler and the matrix, and the agglomeration problem between the filler particles is inevitable. Therefore, with the increase of the filling ratio, the insulating ability of the composite material is further reduced.

**Figure 8:**
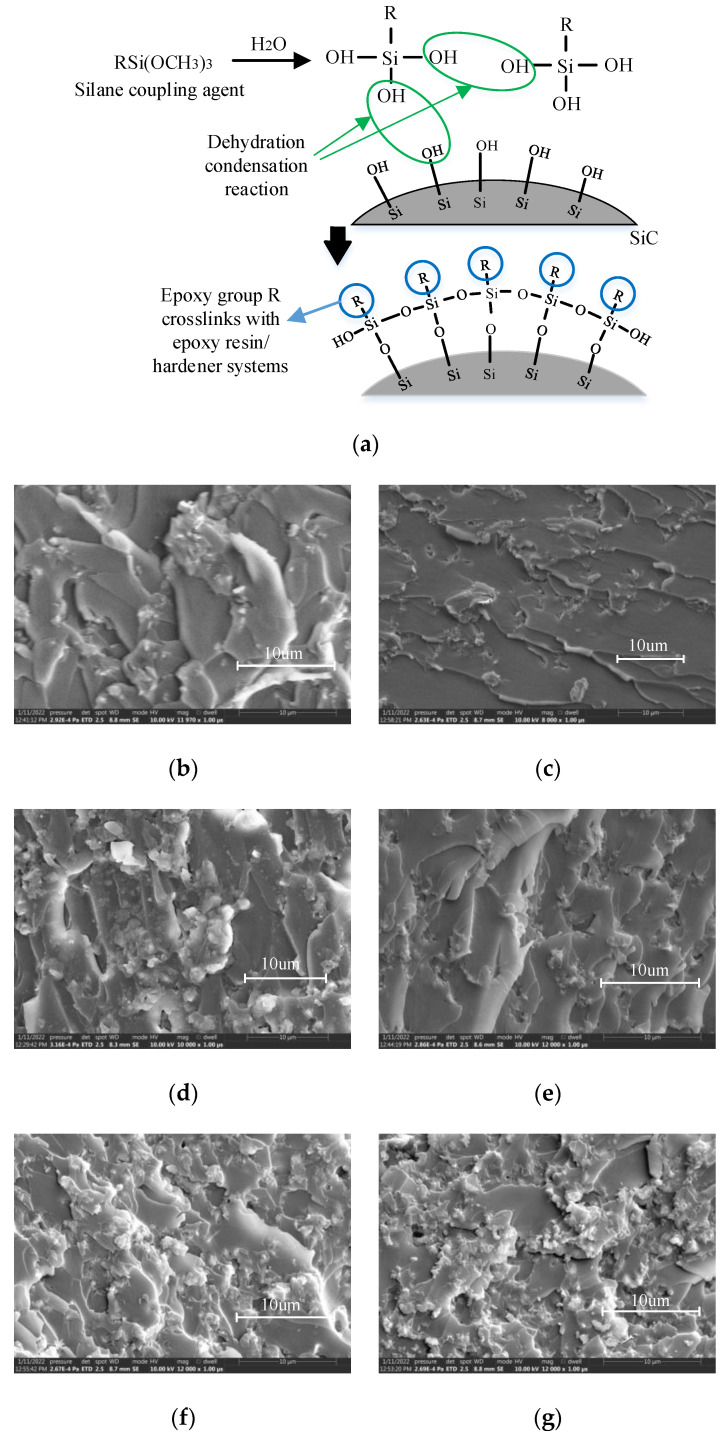
(**a**) Modification of SiC surface by silane coupling agent KH560; (**b**–**g**) Cross-sectional SEM image of SiC/EP composites; (**b**) 10 wt% surface modified SiC/EP; (**c**) 10 wt% surface unmodified SiC/EP; (**d**) 20 wt% surface modified SiC/EP; (**e**) 10 wt% surface unmodified SiC/EP; (**f**) 30 wt% surface modified SiC/EP; (**g**) 30 wt% surface unmodified SiC/EP.

In the experiment, silane coupling agent KH560 was used to modify the SiC surface. The reaction mechanism is shown in Figure 8a. One end of the coupling agent molecule is bonded to the SiC surface, and the other end participates in the cross-linking reaction of the epoxy/curing agent system. The compatibility between the filler and the matrix is improved, the air gap at the interface between the two phases is reduced, and the microscopic defects are reduced, thereby improving the overall breakdown field strength and partial discharge onset voltage of the composite material.

In order to observe the effect of micro-SiC surface particle surface modification on the filler/matrix interface in the composite material more intuitively, a scanning electron microscope (SEM) test was performed on the cross-section of the composite material, as shown in Figure 8b–g.

The SEM results of the cross-section of composite materials showed that there were obvious air gaps between the unmodified filler particles and the matrix, and the proportion of the filler particles exposed outside the epoxy resin matrix at the cross-section was large. After modification, the contact between the filler and the matrix became tighter, the air gap defects were reduced, and the surrounding of the filler was covered by the matrix better than that of the unmodified sample. For unmodified SiC/EP, with the increase of the total filling ratio of SiC, the filler particles appeared agglomeration, which led to the increase of air gaps between the agglomerates and the matrix. Although the modified SiC/EP could improve the bonding performance between the filler and the matrix, when the number of filler particles was too large, filler agglomeration would also occur, the modification effect became more and more limited, and there would still be more air gaps at the interface.

**Figure 9:**
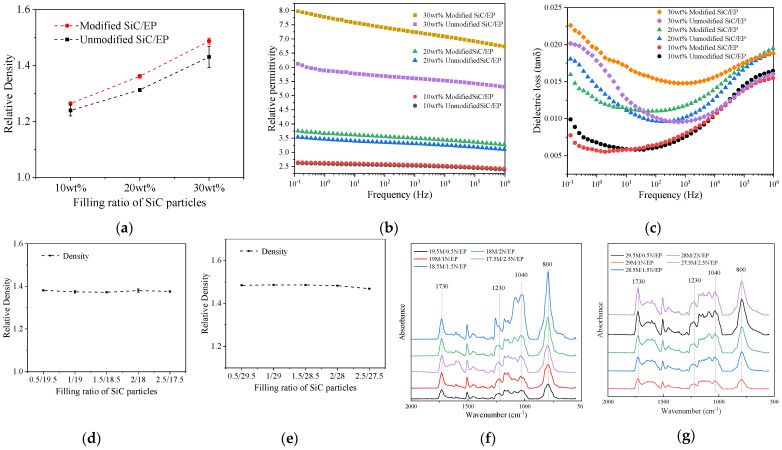
(**a**) Density of SiC/EP composites; (**b**) Dielectric constant of composite materials; (**c**) Composite dielectric loss tangent; (**d**) The total filling ratio of 20 wt%; (**e**) The total filling ratio of 30 wt%; Baseline correction and partial magnification of SiC/EP infrared spectrum: (**f**) FTIR spectra of 20 wt% filled SiC/EP; (**g**) FTIR spectra of 30 wt% filled SiC/EP.

In order to further verify the effect of filler surface modification on improving interfacial bonding properties and reducing air gaps, the density of SiC/EP composites was tested, and the results are shown in Figure 9a.

It can be seen from the results that under the premise of the same curing temperature curve and formulation system, the relative density of the SiC/EP composites modified on the filler surface is significantly higher than that of the unmodified SiC/EP composites.It can be considered that the difference in the density of the composites on the macroscopic scale is caused by the existence of air gaps at the filler/matrix interface. With the increase of the filling ratio, the difference of the relative density of the composites shows an increasing trend.

Compared with the matrix, the SiC filler particles have a much higher dielectric constant, which will inevitably have different effects on the relative dielectric constant of the composite material under different filling ratios, so it is necessary to carry out measurement and characterizatione methods. The dielectric properties of modified SiC/EP composites were tested. The instrument used is a broadband dielectric spectrometer with a measurement range of 10^−1^~10^6^ Hz. The test sample is a wafer with a thickness of 1 mm and a diameter of 50 mm, which meets the dielectric test standards.

The dielectric constants of the composites are shown in Figure 9b under different filling ratios.

With the increase of SiC filling content in the composites, the relative permittivity of the composites increases. Compared with epoxy resin, SiC particles have a higher relative permittivity, and more dipoles were introduced into the composite material [6], and the overall polarization characteristics of the material were enhanced.

The relative permittivity of the secondary modified SiC/EP composites was significantly higher than that of the unmodified SiC/EP composites. And as the filling ratio increased, the numerical difference between the two also increased. This is due to coupling agent molecules, which are polar molecules, are grafted on the surface of SiC during the surface modification process [28]. In the composite material system, with the increase of the filling ratio, the number of polar molecules on the surface of the introduced filler also increased accordingly. This resulted in a larger relative permittivity difference with a higher filling ratio. This trend also verified that polar silane coupling agent molecules were effectively grafted on the surface of the filler.

The dielectric loss tangent of the composite material is shown in Figure 9c. Compared with the obvious regularity of the dielectric constant, the change trend of the dielectric loss tangent value of the composite material was no longer a simple monotonous increase or decrease with the filling ratio or modification. This is because the factors affecting the dielectric loss of the composite materials are not only related to the dielectric constant, but also related to the volume resistivity of the composite materials, the internal state of the solid and other factors. As the filling ratio of SiC particles in the composite material increased, the dielectric loss generally showed an increasing trend. The conductivity of SiC is much larger than that of epoxy resin, and the volume resistivity of the composite material decreases to a certain extent after the introduction of SiC filler. At the same time, combined with the increase of the dielectric constant, the dielectric loss of the composite material showed an increasing trend.

The effect of surface-modified grafted coupling agent molecules on the dielectric loss of the material was much smaller than its effect on the dielectric constant. Under the filling ratio of 10 wt% and 20 wt%, the dielectric loss-frequency curves of the composites have intersecting parts. Under the filling ratio of 30 wt%, the dielectric loss of modified SiC/EP is significantly increased compared with that of unmodified SiC/EP. Based on the analysis of the dielectric properties of the material, the SiC filler will cause the increase of the dielectric constant and dielectric loss of the composite materials, but the increase of the dielectric loss is smaller than the increase of the dielectric constant.

### 3.2. Partial Discharge Initial Voltage of Micro-Nano Compounding Composites

**Figure 10:**
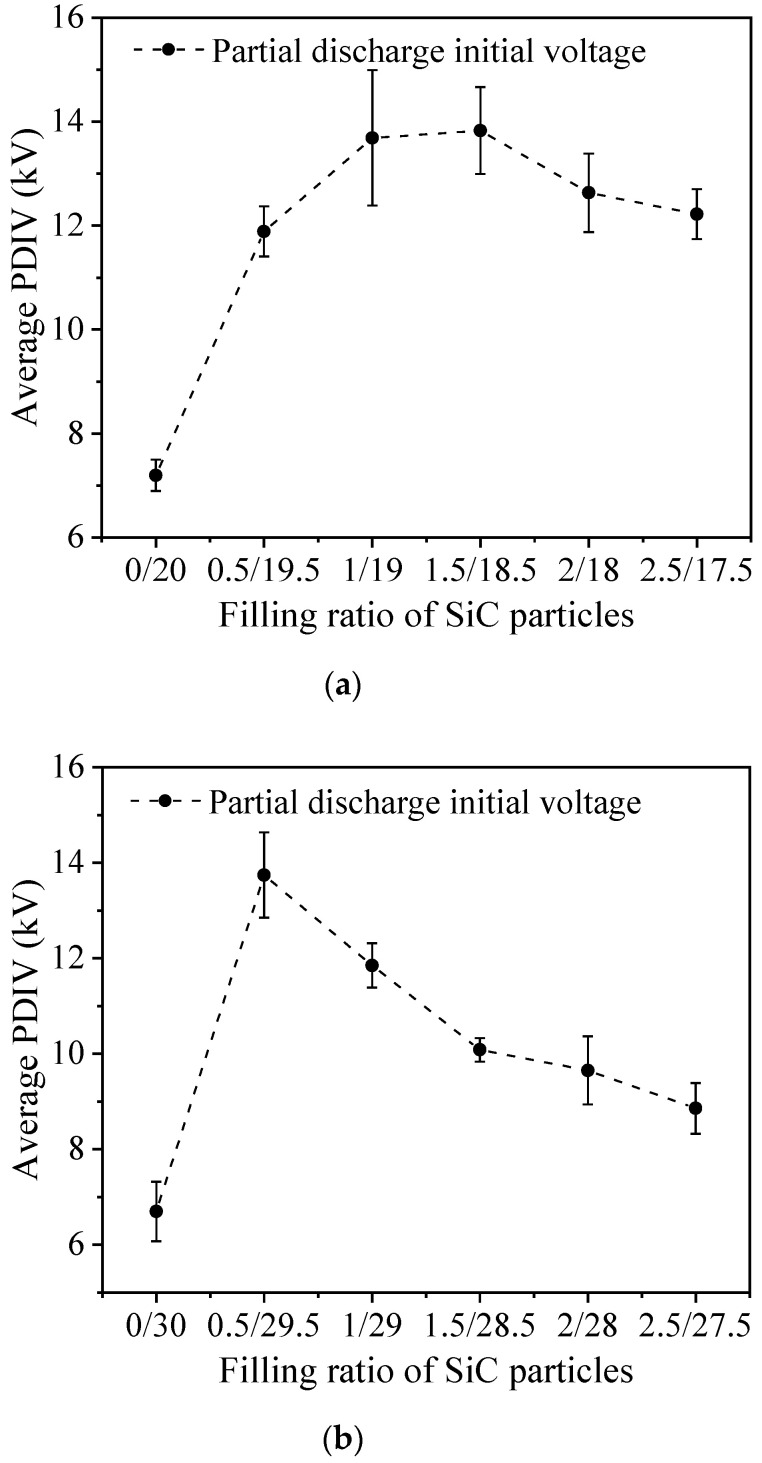
PDIV of micro-nano SiC/EP composites; (**a**) The total filling ratio of 20 wt%; (**b**) The total filling ratio of 30 wt%.

The composite PD sample preparation, electrode burying method, PD test circuit and test method are the same as the previous ones. The partial discharge initial voltage of the composite material was measured under the control background noise of 2 pC, and the partial discharge initial voltage of the composite material under different doping systems was obtained by repeated measurement. The experimental results are shown in Figure 10.

Compared with the SiC/EP composites filled with a single type of microparticles, the PDIV of the materials can be significantly improved after the introduction of nanoparticles into the filling system. And with the increase of the nanoparticle filling ratio, the PDIV of the composites generally showed a trend of first increasing and then decreasing. For SiC/EP composites with a total filling ratio of 20 wt%, the PDIV of the composites gradually increased with the addition of nanoparticles. The PDIV of the composite reaches its maximum value when the micro-particle to nano-particle filling ratio is 18.5M/1.5N, which is 92.16% higher than that of the pure micro-particle filled composite. As the proportion of nanoparticles continued to increase, PDIV showed a slight downward trend. For SiC/EP composites with a total filling ratio of 30 wt%, adding nanoparticles could also improve the PDIV of the composites. The optimal filling ratio of microparticles and nanoparticles is 29.5M/0.5N. The partial discharge initial voltage of the composite material without nanoparticles was increased by 105.12%. When the filling ratio of nanoparticles was further increased, the PDIV also showed a downward trend.

The density of the micro-nano composite SiC/EP composite was measured, and the measurement results are shown in Figure 9d,e.

It can be seen from Figure 9d,e that, unlike the experimental results of surface modification of SiC fillers, the compounding of micro-nano does not have much effect on the relative density of composites. When the content of nanoparticles increased, the density of the composites changed little. For SiC/EP with a total filling of 30 wt%, when the content of nanoparticles was 2.5 wt%, the relative density of composite materials decreased slightly, which might be caused by the agglomeration of particles due to the excessive content of nanoparticles.

Fourier infrared spectroscopy tests were performed on the composites. The results showed that after adding nano-SiC particles to the SiC/EP composites, the PDIV variation trend of the composites with different filling ratios showed a strong correspondence with the changes of the characteristic peak intensity of the infrared spectrum of composites.

The infrared transmission spectrum was converted into absorption spectrum, baseline correction and local amplification were performed, and the relative sizes of absorption peaks were intuitively compared, which can be seen in Figure 9f,g.

The wave number of 800 cm^−1^ corresponds to the C-N single bond, and the stronger the absorption peak, the higher the concentration of amide, indicating that the reaction between the tertiary amine accelerator and the acid anhydride curing agent is more sufficient. When the filling amount of nanoparticles was changed, the degree of reaction between the tertiary amine accelerator and the acid anhydride curing agent changed, which also verifies that the filling of nanoparticles has a significant effect on the curing reaction of the epoxy resin matrix of the composite material. 1230 cm^−1^ and 1170 cm^−1^ are the absorption bands of esters, and 1040 cm^−1^ is the absorption band of primary alcohols. The higher the crosslinking degree of epoxy resin after curing, the stronger the absorption peak of the corresponding band [29].

**Figure 11:**
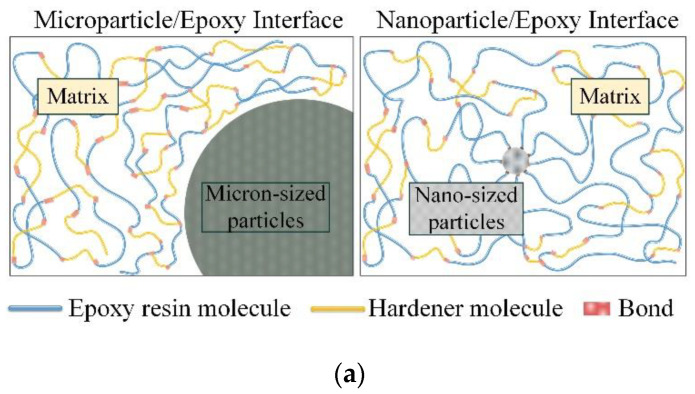

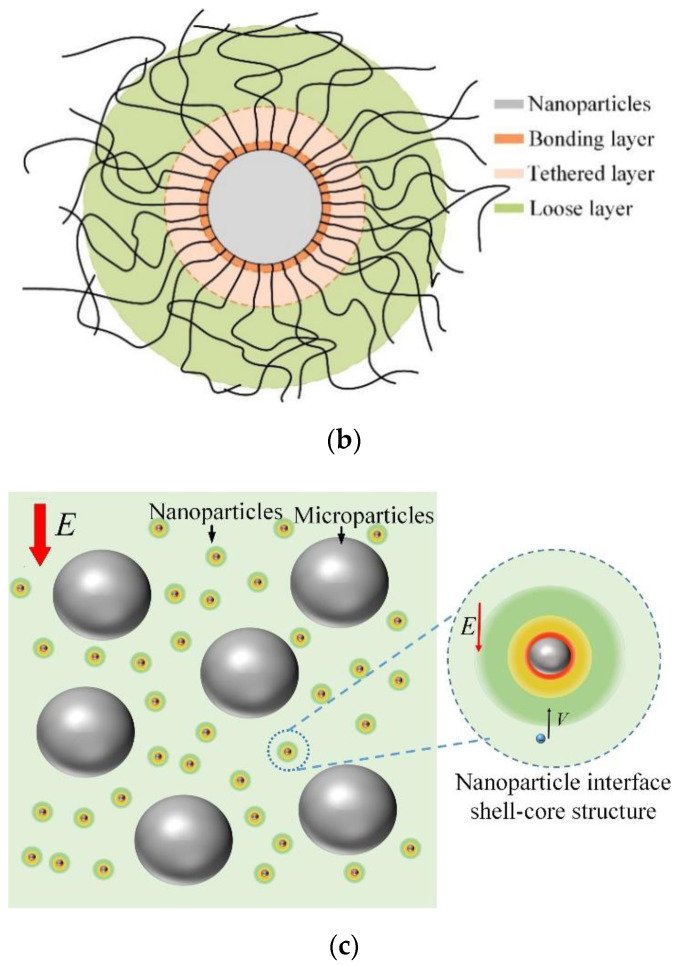
(**a**) Schematic diagram of filler/matrix interface; (**b**) Shell-core structure of nanoparticles and matrix; (**c**) Schematic diagram of micro/nanoparticle composites.

When the added amount was appropriate, the SiC nanoparticles were uniformly dispersed, and the surface of the SiC nanoparticles underwent bonding reaction with the epoxy matrix during the curing reaction, which enhanced the cross-linking degree of the epoxy resin, as shown in Figure 11a. However, when the filling ratio of nano-SiC particles was too large, the nanoparticles would agglomerate due to the large surface potential energy [30], the equivalent radius of the agglomerates would increase, and a large number of void defects would be formed inside, resulting in a decrease in the epoxy crosslinking degree.

Nanoparticle has a small radius and a large specific surface area. As the particle size decreases, the ratio of the number of surface atoms to the total number of atoms in the particle increases. The atoms located on the surface are insufficiently coordinated, and there are a large number of dangling bonds, making them highly reactive and easy to bond with other atoms. In the process of mixing and curing with epoxy resin, the unsaturated bonds on the surface of nanoparticles are directly bonded with epoxy groups, forming an interactive structure on the surface of the particles [31,32], which can be regarded as nano size effects or interface effects of particles. The bonding and cross-linking structure of polymer chains at the interface have a significant impact on the carrier trap density and polarization of composites. Hierarchical structures similar to “shell-core” are often employed to explain these effects [32,33].

One end of the organic polymer macromolecule is tightly connected with the nanoparticle surface by covalent bond, van der Waals force or electrostatic force, resulting in a layered structure at the interface of the nanoparticle, as shown in Figure 11b. The innermost layer has the strongest force, which is called the bonding layer; the outermost layer has a high randomness in the arrangement of polymers, which is called the loose layer; the part between the two is called the tethered layer, and the molecular arrangement of the tethered layer is affected by the influence of the bonding area, the bonding strength is between the bonding layer and the loose layer. In addition, the interface part can also be defined as bonding area, transition area, and normal area from inside to outside [34].

Due to the change of the bonding strength at the interface, the carriers need to obtain enough energy to cross the potential barrier when transitioning between layers [12]. When the mean free path of carriers is too small, the electric field cannot gain enough energy to pass through the well barrier between the binding layer and the bonding layer, thus trapped in the bound layer. When the nanoparticles are uniformly dispersed inside the composite materials, the existence of nano-interface introduces deep traps to capture the freely moving carriers, which reduces the average mobility and energy of the carriers inside the overall medium, thereby reducing the impact ionization inside the composite materials [16]. At the macroscopic level, it is manifested as an increase in the PDIV of composite materials.

## 4. Conclusions

In this paper, aiming at improving the partial discharge initial voltage of SiC/EP composite insulating materials, the effect of surface modification of micro-SiC particles and micro-nano SiC fillers on improving the PDIV of composite materials were investigated. The SiC/EP composite material was prepared, a partial discharge experimental test platform was built to measure the PDIV, and dielectric properties of the composite material were characterized. Density, SEM, Fourier infrared spectroscopy and other methods were applied to explore the internal mechanism, the conclusions are as follows.

(1) The surface modification of micron SiC particles by the method of secondary treatment of KH560 coupling agent after alkaline washing could improve the bonding degree between fillers and epoxy polymer matrix, reduce interfacial air gap defects, and increase the density of composite materials. Thus, the PDIV of composite materials was increased. After the SiC surface was modified with silane coupling agent, the polar groups of coupling agent molecules were introduced into the SiC/EP composite materials, thereby increasing the dielectric constant of composite materials.
(2) The compounding of micro-nano SiC particles can significantly improve the PDIV of SiC/EP composites. Due to the large specific surface area of the nanoparticles, a nanoscale effect was produced, and a shell-core structure was generated inside the composite materials. The interface between the shells raised the barrier height of carrier migration and introduced deep traps, thus hindering the movement of carriers in the composite materials, and the PDIV of composite materials finally increased.
(3) Under different total filling ratios of fillers, the optimal ratio of micro-nano particles is different. For 20 wt% SiC/EP composites, the optimal ratio of micro-nanoparticles is 18.5 wt% micron particles and 1.5 wt% nanoparticles, and for 30 wt% SiC/EP composites, the optimal ratio of micro-nanoparticles is 29.5 wt% micron particles and 0.5 wt% nanoparticles.

## Figures and Tables

**Table 1:** Experimental materials used in the experiments.

Number	Name	Material Properties	Manufacturer
1	Epoxy resin E51	Epoxy value 0.48~0.54 eq/100 g Viscosity	Shanghai Xiongrun Resin Co.
2	Methylcyclohexene-1,2-dicarboxylic anhydride (curing agent)	Analysis pure	TCI (Shanghai) Kasei Industrial Development Co.
3	2,4,6-Tris(dimethylaminomethyl)phenol (accelerator)	Analysis pure	TCI (Shanghai) Kasei Industrial Development Co.
4	Silicon carbide (β-SiC)	Particle size: 1.5 μm	Qinhuangdao Yino Company
5	Silicon carbide	Particle size: 50 nm	Qinhuangdao Yino Company
6	γ-(2,3-epoxypropoxy) propyltrimethoxysilane (KH-560)	Purity >98%	Aladdin Technology Shanghai
7	Anhydrous ethanol	Analysis pure	Beijing Inokai Technology Co.
8	NaOH	≥99%	Beijing Inokai Technology Co.

**Table 2:** Experimental apparatus.

Number	Name	Model	Manufacturers
1	Blast Drying Ovens	101-2AB	Tianjin Teste Instruments
2	Mechanical Mixers	Nanostar 7.5 Digital	IKA Germany
3	Electronic Scales	BSM-223	Beijing Xijie Balance Instruments
4	Infrared Spectroscopy	Invenio S	BRUKER Corporation, USA
6	Constant Temperature Oil Baths	DZKW-4	Beijing Zhongxingweiye Instruments
7	Electronic Density Tester	MH-300a	Un Long Corporation
8	Broadband Dielectric Spectroscopy	Concept 80	Novocontrol, Germany
9	Scanning Electron Microscope	Quanta 200 Feg	Fei USA, Inc.
10	Centrifuges	MD550A	Shanghai Merrick Instruments Co.
11	Magnetic Stirrers	MS-H-Pro+	Beijing DRAGON LAB Company

**Table 3:** SiC/EP composites filled with micro-nano particles.

Number	Mass Fraction of m-SiC	Mass Fraction of n-SiC	Serial Number
1	19.5 wt%	0.5 wt%	19.5M/0.5N/SiC/EP
2	19 wt%	1 wt%	19M/1N/SiC/EP
3	18.5 wt%	1.5 wt%	18.5M/1.5N/SiC/EP
4	18 wt%	2 wt%	18M/2N/SiC/EP
5	17.5 wt%	2.5 wt%	17.5M/2.5N/SiC/EP
6	29.5 wt%	0.5 wt%	29.5M/0.5N/SiC/EP
7	29 wt%	1 wt%	29M/1N/SiC/EP
8	28.5 wt%	1.5 wt%	28.5M/1.5N/SiC/EP
9	28 wt%	2 wt%	28M/2N/SiC/EP
10	27.5 wt%	2.5 wt%	27.5M/2.5N/SiC/EP

**Table 4:** Equipment for partial discharge testing.

Number	Name	Model	Manufacturer
1	Signal generator	AFG3022C	Tektronix Co. LTD
2	Voltage amplifier	AMPS	Matsusada Co. LTD
3	Oscilloscope	MSO44	Tektronix Co. LTD
4	Coupling capacitor	TAWF-50kV/800pF	Wuhan Xigao Electric Co., LTD
5	Digital PD meter	MPD 600	OMICRON Co. LTD
6	Fiber optic bus controller	MCU502	OMICRON Co. LTD
7	Charge calibrator	CAL 542	OMICRON Co. LTD
8	Detecting impedance	CPL 542	OMICRON Co. LTD
9	High voltage probe	PVM-4	North star Co. LTD

**Table 5:** Parameters of SiC filler and epoxy resin matrix in composites.

Material	Relative Permittivity	Conductivity (S/m)
SiC particles	10	$4.14\times 10^{-9}\times e^{2.72\times 10^{-7}E}$
Epoxy resin	2.3	$1\times 10^{-15}\times e^{9.4\times 10^{-8}E}$

## Data Availability

The data presented in this study are available on request from the corresponding author.
